# Obesity surgery and serious psychiatric conditions, lessons learned from 5 patients: A case report

**DOI:** 10.1097/MD.0000000000048177

**Published:** 2026-03-27

**Authors:** Oussama Shibly, Maher Salloum, Philippe Attieh, Pedro Yammine, Mustafa Allouch

**Affiliations:** a Department of General Surgery, University of Balamand, Beirut, Lebanon; b Department of Orthopedics surgery, Saint George University Hospital, Beirut, Lebanon; c Chairman of Surgery Department, Nini Hospital, Tripoli, Lebanon.

**Keywords:** bariatric surgery, ethical considerations in bariatric surgery, multidisciplinary evaluation in obese psychiatric patients, obesity and mental health, postoperative complications, psychiatric relapse post-bariatric surgery, serious psychiatric disorders

## Abstract

**Rationale:**

Obesity and severe psychiatric disorders, including schizophrenia, bipolar disorder, and major depressive disorder, frequently coexist, with obesity prevalence nearly twice that in the general population. This association is driven by antipsychotic-induced metabolic effects, sedentary behavior, and poor dietary habits. Despite the proven efficacy of bariatric surgery for morbid obesity, its application in patients with serious psychiatric illness remains controversial due to concerns regarding treatment adherence, psychiatric relapse, and decision-making capacity.

**Patient concerns:**

Five patients with established severe psychiatric disorders (schizophrenia, bipolar disorder, and severe depression) presented with morbid obesity requiring surgical management. Concerns included poor adherence to medical recommendations, vulnerability to psychiatric decompensation, and limited preoperative psychiatric evaluation.

**Diagnoses:**

All patients were diagnosed with morbid obesity in the setting of chronic severe psychiatric illness. Postoperatively, several patients developed complications, including psychiatric relapse (psychotic or mood episodes), somatic symptomatology without an identifiable organic cause, and surgical complications.

**Interventions:**

Patients underwent various bariatric procedures. However, preoperative psychiatric assessment was insufficient, and structured postoperative psychiatric follow-up was lacking across all cases.

**Outcomes:**

Although significant weight loss and metabolic improvement were achieved in all patients, outcomes were overshadowed by major adverse events. These included psychiatric relapses, treatment noncompliance, functional somatic symptoms, surgical complications, and one completed suicide.

**Lessons:**

Bariatric surgery in patients with severe psychiatric disorders carries substantial risk when not managed within a multidisciplinary framework. Thorough preoperative psychiatric evaluation and continuous postoperative mental health support are essential to optimize outcomes. Surgical candidacy should be based on individualized functional stability rather than diagnosis alone, and such procedures should be limited to specialized centers capable of addressing complex psychiatric and medical needs.

## 1. Introduction

Obesity and psychiatric disorders represent 2 significant public health challenges worldwide. According to the World Health Organization, more than 1 billion people globally are affected by obesity – including 650 million adults, 340 million adolescents, and 39 million children representing roughly 1 in 8 individuals worldwide. Similarly, serious psychiatric disorders such as severe depression, bipolar disorder, and schizophrenia affect ~970 million people globally and contribute to nearly 14% to 16% of the global disease burden, as reported by the Global Burden of Disease Study 2019.^[1]^ These rising rates highlight the urgency of addressing the clinical and ethical complexities associated with their coexistence.

The co-occurrence of obesity and serious psychiatric conditions presents a common and complex clinical scenario with unique challenges. Epidemiological studies show that individuals with severe mental illness are 2 to 3 times more likely to develop obesity than the general population, with reported prevalence rates of 40% to 60% among those with schizophrenia and up to 50% among those with bipolar disorder.^[2]^ This overlap stems largely from the metabolic side effects of second-generation antipsychotics – particularly clozapine and olanzapine – which can induce an average 4 to 7 kg weight gain within 10 weeks of treatment, alongside insulin resistance, dyslipidemia, and metabolic syndrome.^[3]^ Additionally, unhealthy lifestyle behaviors, poor diet, and sedentary habits – often exacerbated by psychiatric symptoms such as low motivation and anhedonia – contribute to progressive weight gain and worsened medical outcomes.^[3]^ Beyond biological mechanisms, individuals with serious psychiatric conditions face substantial systemic barriers to obesity treatment. These include stigma and discrimination in healthcare settings, fragmented coordination between psychiatric and medical services, limited access to multidisciplinary programs, and medication-induced metabolic side effects that compound existing risks.^[2,3]^ Applying a biopsychosocial framework helps conceptualize these interactions: biological vulnerability (antipsychotic-related weight gain and endocrine dysfunction), psychological impediments (reduced insight, cognitive deficits), and social barriers (stigma, socioeconomic disadvantage, and healthcare fragmentation) synergistically limit treatment efficacy and perpetuate poor outcomes.

Bariatric surgery remains one of the most effective interventions for severe obesity, achieving sustained weight loss and improvement in metabolic comorbidities. However, its implementation in patients with serious psychiatric illnesses raises clinical, ethical, and logistical concerns, particularly regarding adherence, relapse risk, and postoperative psychosocial stability.^[1]^ Evidence on long-term outcomes in this subgroup remains limited, with most reports focusing on short-term safety or weight loss metrics rather than psychiatric or ethical dimensions.

Therefore, this case series aims to address this gap by reviewing real-world experiences from a single center involving 5 patients with severe psychiatric conditions who underwent bariatric surgery. The objective is to identify modifiable risk factors associated with adverse outcomes, highlight the importance of multidisciplinary pre- and postoperative evaluation, and contribute to the ongoing debate surrounding the ethical justification and clinical management of bariatric surgery in psychiatric populations.

## 2. Materials and methods

### 2.1. Study design and setting

This study was designed as a retrospective case series conducted at Nini Hospital, Tripoli, Lebanon. The review included all patients with a documented diagnosis of a serious psychiatric disorder who underwent bariatric surgery between January 2015 and December 2022. The study followed the STROBE (Strengthening the Reporting of Observational Studies in Epidemiology) guidelines for observational research. Medical and surgical records were reviewed to assess postoperative outcomes, psychiatric stability, and complications.

### 2.2. Case selection and eligibility criteria

Cases were identified through hospital electronic medical records and surgical registries using International classification of diseases (ICD)-10 diagnostic codes related to serious psychiatric disorders, including F20–F29 (schizophrenia spectrum disorders), F31 (bipolar affective disorder), and F32–F33 (major depressive disorder). Inclusion criteria were:

1. Adults (≥18 years) with morbid obesity defined as Body mass index (BMI) ≥ 40 kg/m^2^, or BMI ≥ 35 kg/m^2^ with at least 1 obesity-related comorbidity (e.g., diabetes mellitus, hypertension).
2. Confirmed diagnosis of a serious psychiatric condition by a psychiatrist prior to surgery.
3. Underwent a primary bariatric procedure (laparoscopic sleeve gastrectomy, Roux-en-Y gastric bypass, or adjustable gastric banding).

### 2.3. Exclusion criteria included

1. Patients undergoing revisional or emergency bariatric surgery.
2. Incomplete psychiatric or surgical records.
3. Lack of postoperative follow-up beyond 12 months.

### 2.4. Data collection and variables

Data extraction was performed manually by 2 independent investigators using a standardized data abstraction form. Discrepancies were resolved by consensus. Extracted variables included:

- Demographic data: age, sex, BMI, and comorbidities (e.g., diabetes, hypertension).
- Psychiatric characteristics: diagnosis, duration of illness, treatment regimen, and preoperative psychiatric stability.
- Surgical details: type of bariatric procedure, intraoperative findings, and length of hospital stay.
- Postoperative outcomes:
  ○ Weight loss (percentage of excess weight loss [% excess weight loss] at 6 and 12 months).
  ○ Complications, classified according to the Clavien–Dindo system.
  ○ Psychiatric outcomes, including relapse, hospitalization, suicide attempt, or change in medication.
  ○ Adherence to psychiatric and surgical follow-up, evaluated through chart documentation and recorded clinic attendance.

Follow-up duration ranged from 12 to 60 months, depending on case availability. Psychiatric relapse was defined as a clinically significant exacerbation of psychotic, mood, or affective symptoms requiring medication adjustment or psychiatric hospitalization.

### 2.5. Data analysis

Given the small sample size and descriptive design, quantitative data were summarized using descriptive statistics (mean, range, and percentage). Qualitative analysis was performed to identify recurring themes related to adverse outcomes, including treatment non-adherence, psychosocial instability, and system-level factors contributing to complications. This thematic synthesis allowed for the identification of modifiable risk factors and guided the interpretation of clinical lessons learned.

### 2.6. Ethical considerations

The study protocol was reviewed and approved by the Ethics Committee of Nini Hospital (Reference: NNH-SUR-2022-015). All procedures were conducted in accordance with the principles of the Declaration of Helsinki (2013 revision). Data confidentiality was maintained by anonymizing patient identifiers and using encrypted files accessible only to the research team. Since this was a retrospective chart review, the requirement for additional written consent was waived by the institutional review board; however, all patients had provided prior informed consent for their anonymized data to be used in academic reporting. Patient information was de-identified in compliance with HIPAA-equivalent privacy standards to ensure confidentiality.

## 3. Case presentation

### 3.1. Case 1

#### 3.1.1. Demographics

A 32-year-old female, BMI 43 kg/m^2^, presented requesting bariatric surgery for “overweight” (Table 1).

**Table 1:** Summary of the cases.

Case	Age (yr)	Sex	Psychiatric diagnosis	Surgery type	Preoperative BMI (kg/m^2^)	Follow-up (mo)	%TBWL	Major postoperative events	Psychiatric relapse	Outcome
1	32	F	Undiagnosed psychotic disorder (later schizophrenia)	Lap-band	43	12	18%	Severe abdominal pain without organic cause; lap-band removal	Yes	Stable after device removal
2	42	F	Schizophrenia	Lap-band	42	60	20%	Severe reflux and recurrent pneumonia	No	Lost to follow-up
3	32	F	Schizophrenia	Omega loop bypass	36	48	35%	Hallucinations; possible medication malabsorption	Yes	Bypass reversal; lost to follow-up
4	50	F	Bipolar disorder	Sleeve gastrectomy	35	18	32%	Recurrent unexplained epigastric pain	Yes	Suicide at 18 mo
5	25	F	Severe depression with OCD traits	Lap-band	75	36	60%	Boerhaave-like rupture; multiple reoperations	No	Recovered; weight regained

#### 3.1.2. Preoperative assessment

No documented comorbidities or psychiatric evaluation at baseline; appeared mentally stable during consultation.

#### 3.1.3. Surgical procedure

Underwent laparoscopic adjustable gastric banding without intraoperative complications.

#### 3.1.4. Postoperative course

Within 6 to 8 hours post-surgery, the patient reported severe abdominal pain despite normal imaging and labs. Pain improved only with opioids and resolved by postoperative day 4.

#### 3.1.5. Outcomes

Approximately 1 month later, the patient returned requesting band removal due to “foreign body sensations.” After removal, she was later diagnosed with schizophrenia. At 12-month follow-up, her % total body weight loss (TBWL) was 18%.

### 3.2. Case 2

#### 3.2.1. Demographics

A 42-year-old woman with schizophrenia, type 2 diabetes, and BMI 42 kg/m^2^ (Table 1).

#### 3.2.2. Preoperative

Cleared for surgery by her psychiatrist as clinically stable on antipsychotic therapy.

#### 3.2.3. Surgical procedure

Laparoscopic gastric banding was performed uneventfully.

#### 3.2.4. Postoperative course

Achieved significant improvement in glycemic control and %TBWL of 20% within 12 months. At 5 years, she developed severe gastroesophageal reflux and recurrent aspiration pneumonia.

#### 3.2.5. Outcomes

The patient refused band deflation or removal despite counseling and was eventually lost to follow-up. No documented psychiatric relapse during follow-up.

### 3.3. Case 3

#### 3.3.1. Demographics

A 32-year-old woman with schizophrenia and diabetes, BMI 36 kg/m^2^ (Table 1).

#### 3.3.2. Preoperative assessment

Deemed stable by an overseas psychiatrist. Understood surgical risks but insisted on bypass despite medical advice for a sleeve procedure.

#### 3.3.3. Surgical procedure

Underwent Omega loop gastric bypass (biliopancreatic limb 1.5 m).

#### 3.3.4. Postoperative course

Achieved %TBWL of 35% and normalization of glycemia within 12 months. After 3 years, developed hallucinations and reported seeing undigested pills in stool.

#### 3.3.5. Outcomes

Unclear adherence to medication; bypass was reversed following psychiatric consultation. The patient left the country and was lost to follow-up.

### 3.4. Case 4

#### 3.4.1. Demographics

A 50-year-old woman with bipolar disorder and insulin-dependent diabetes, BMI 35 kg/m^2^ (Table 1).

#### 3.4.2. Preoperative assessment

Cleared for surgery by her psychiatrist; insulin requirements were high preoperatively.

#### 3.4.3. Surgical procedure

Underwent laparoscopic sleeve gastrectomy without complication.

#### 3.4.4. Postoperative course

Within 6 months, achieved 32% TBWL, euglycemia, and stopped insulin therapy. Later developed chronic epigastric pain despite normal diagnostic tests.

#### 3.4.5. Outcomes

At 18 months post-surgery, the patient died by suicide. No surgical pathology was found at previous admissions.

### 3.5. Case 5

#### 3.5.1. Demographics

A 25-year-old female with morbid obesity (BMI 75 kg/m^2^) and unrecognized psychiatric symptoms (obsessive and impulsive traits; Table 1).

#### 3.5.2. Preoperative assessment

No psychiatric consultation obtained due to personal acquaintance with the surgeon.

#### 3.5.3. Surgical procedure

Laparoscopic gastric banding was performed successfully.

#### 3.5.4. Postoperative course

Within 3 years, achieved 60% TBWL, but developed recurrent vomiting and binge-eating behavior.

#### 3.5.5. Outcomes

Presented with Boerhaave-like syndrome due to spontaneous esophageal rupture requiring multiple abdominal and thoracic interventions. Recovered but regained all weight within 6 months. Later diagnosed with severe depression and compulsive behaviors.

## 4. Discussion

### 4.1. Prevalence and psychiatric associations

Obesity and serious psychiatric conditions, including schizophrenia, bipolar disorder, and major depression, frequently co-occur, with evidence suggesting a bidirectional relationship. Individuals with severe mental illness exhibit an obesity prevalence nearly double that of the general population, largely due to metabolic side effects of psychotropic medications and unhealthy lifestyle behaviors. This overlap contributes to higher morbidity and premature mortality, reducing life expectancy by 10 to 20 years.^[4]^

Beyond these epidemiologic figures, the psychological burden of obesity itself can exacerbate psychiatric distress, generating a cycle of metabolic and affective deterioration.^[5-7]^ While some studies suggest obesity may confer a paradoxical protective effect against suicide in men, others report heightened suicidal ideation among women,^[8]^ highlighting the need to contextualize these findings within sex-specific and illness-specific frameworks. This nuanced relationship underscores the importance of targeted preoperative psychiatric assessment before bariatric surgery.

### 4.2. Surgical outcomes in psychiatric populations

Bariatric surgery remains the most effective long-term therapy for severe obesity, achieving durable weight loss and remission of metabolic comorbidities.^[9,10]^ Yet, outcomes among patients with serious psychiatric illness are heterogeneous. Although some series demonstrate comparable weight loss and metabolic improvements to the general population,^[11,12]^ psychiatric relapse, postoperative non-adherence, and psychosomatic symptoms are not uncommon, as illustrated by the present cases. These complications may stem from impaired insight, medication malabsorption (as in case 3), or inadequate postoperative monitoring.

Recent reviews emphasize that stable psychiatric patients can safely undergo bariatric surgery when managed within structured multidisciplinary programs that include psychiatrists, psychologists, and clinical pharmacists.^[11,13]^ Integration of mental health professionals into surgical teams enhances adherence, early detection of relapse, and optimization of psychotropic medication regimens. Conversely, lack of coordinated psychiatric input – as seen in several of our cases – was associated with adverse outcomes, including suicide and recurrent psychosomatic pain.

### 4.3. Ethical and clinical considerations

Ethical discourse surrounding bariatric surgery in psychiatric populations revolves around autonomy, capacity, and distributive justice. Historically, psychotic or affective disorders were considered relative contraindications to surgery.^[2]^ However, contemporary frameworks emphasize functional stability and decision-making capacity rather than diagnostic labels.^[14]^ Denying surgery solely on psychiatric grounds may constitute discrimination, particularly when obesity itself poses greater health risks.

From a clinical ethics perspective, informed consent must encompass discussion of potential psychiatric destabilization, altered drug absorption, and postoperative behavioral demands. Ethical practice also mandates equitable access to surgery within systems capable of providing psychiatric aftercare, rather than exclusion based on diagnosis. A preoperative capacity assessment and shared-decision model should therefore be standard of care for this vulnerable group.

### 4.4. Study limitations and future directions

Several limitations constrain the interpretation of this case series. The small sample size and single-center design limit external validity. Selection bias may have occurred, as only cases with documented adverse events were captured, potentially overestimating complication rates. Recall bias and incomplete chart documentation further restrict causal inference. In addition, heterogeneity in psychiatric diagnoses, lack of standardized evaluation tools, and inconsistent follow-up hindered comparability. Future prospective, multicenter studies should employ validated psychiatric and quality-of-life measures, structured perioperative mental health pathways, and analytic techniques such as propensity score matching to minimize confounding and strengthen causal conclusions.^[15]^

### 4.5. Practical recommendations

The findings from our cases support a shift from generalized recommendations to case-linked, evidence-informed protocols. For instance, in patients undergoing gastric bypass (case 3), postoperative psychiatric destabilization related to altered drug absorption suggests the need for systematic medication bioavailability monitoring involving clinical pharmacists. Likewise, following the suicide observed in case 4, structured psychiatric follow-up – comprising quarterly evaluations, standardized depression screening, and crisis response planning – should be embedded in postoperative care.

A practical multidisciplinary model would include:

- Preoperative: psychiatric stability verification ≥6 months, cognitive and adherence assessment, and shared-decision documentation;
- Postoperative (0–12 months): monthly psychological sessions, medication review at 3-month intervals, and coordinated nutrition-psychiatry follow-up;
- Long-term (>1 year): annual psychosocial reassessment and targeted interventions for emerging depression or substance use.

Recent consensus statements reinforce the role of such integrated care pathways in optimizing both surgical and psychiatric outcomes.^[16]^ Implementing standardized mental health protocols in bariatric centers would align practice with ethical imperatives while mitigating preventable complications.

## 5. Conclusion

This case series highlights the complex interplay between psychiatric stability and metabolic outcomes following bariatric surgery. The 5 cases collectively demonstrate that while weight reduction and metabolic improvements can be achieved, the absence of structured multidisciplinary care may lead to devastating psychiatric and somatic complications – including relapse, psychosomatic syndromes, and suicide. These outcomes underscore that psychiatric oversight is not supplementary but central to the success of obesity surgery in this population.

Beyond reaffirming the efficacy of bariatric procedures, these findings reveal critical gaps in perioperative management. The observed complications arose not from the surgical techniques themselves, but from systemic oversights – chiefly the failure to ensure psychiatric readiness, postoperative monitoring, and coordinated care. Therefore, improving outcomes for psychiatric patients undergoing bariatric surgery requires transforming lessons learned from adverse experiences into actionable clinical protocols.

### 5.1. Clinical practice recommendations

Based on these cases and existing evidence, the following measures are essential for centers performing bariatric surgery in patients with serious psychiatric illness:

- Preoperative phase
  ○ Conduct a comprehensive psychiatric and cognitive assessment, including evaluation of insight, adherence history, and decision-making capacity.
  ○ Require a minimum of 6 months of psychiatric stability before surgery, verified by a treating psychiatrist.
  ○ Implement shared-decision counseling sessions involving the surgeon, psychiatrist, and patient to document understanding of postoperative expectations and risks.
- Perioperative and early postoperative phase
  ○ Ensure on-site or on-call psychiatric support within the bariatric center for crisis management.
  ○ Integrate clinical pharmacists to monitor potential medication malabsorption and adjust psychotropic regimens.
  ○ Schedule multidisciplinary follow-up meetings (surgery, psychiatry, psychology, nutrition) at least monthly during the first 6 postoperative months.
- Long-term follow-up phase
  ○ Establish quarterly psychiatric reviews during the first postoperative year, then annually thereafter.
  ○ Facilitate patient support groups for ongoing psychosocial adjustment and adherence reinforcement.
  ○ Maintain a centralized tracking registry for psychiatric and metabolic outcomes to identify early warning patterns of relapse or self-harm risk.

These measures should be considered minimum standards for institutional eligibility to perform bariatric procedures in patients with major psychiatric disorders. Centers lacking such infrastructure should refer patients to specialized units capable of providing integrated care.

### 5.2. Future directions

To strengthen the evidence base, prospective multicenter studies and randomized controlled trials are needed to evaluate the safety and efficacy of these structured protocols. Key priorities include:

- Quantifying the impact of integrated psychiatric follow-up on postoperative relapse rates,
- Determining optimal pharmacological monitoring strategies for altered drug absorption post-bypass, and
- Developing validated preoperative psychiatric screening tools tailored for bariatric populations.

In summary, these cases reaffirm that bariatric surgery in psychiatric populations is feasible but only when psychiatric evaluation, perioperative supervision, and long-term follow-up are systematically embedded within care delivery. The integration of mental health into surgical pathways is not merely ethical – it is lifesaving.

## Acknowledgments

The authors would like to acknowledge NINI hospital and the Department of Biomedical Sciences, Faculty of Medicine and Medical Sciences, University of Balamand, Al-Koura, Lebanon, for their support.

## Author contributions

**Conceptualization:** Mustafa Allouch.

**Data curation:** Mustafa Allouch.

**Investigation:** Mustafa Allouch.

**Project administration:** Mustafa Allouch.

**Supervision:** Mustafa Allouch.

**Validation:** Maher Salloum, Mustafa Allouch.

**Visualization:** Maher Salloum, Mustafa Allouch.

**Writing – original draft:** Oussama Shibly, Maher Salloum, Philippe Attieh, Pedro Yammine, Mustafa Allouch.

**Writing – review & editing:** Oussama Shibly, Maher Salloum, Philippe Attieh, Pedro Yammine, Mustafa Allouch.
